# A Biological Age Model Designed for Health Promotion Interventions: Protocol for an Interdisciplinary Study for Model Development

**DOI:** 10.2196/19209

**Published:** 2020-10-26

**Authors:** Karina Louise Skov Husted, Mathilde Fogelstrøm, Pernille Hulst, Andreas Brink-Kjær, Kaj-Åge Henneberg, Helge Bjarup Dissing Sorensen, Flemming Dela, Jørn Wulff Helge

**Affiliations:** 1 Xlab, Center for Healthy Aging Department of Biomedical Sciences University of Copenhagen Copenhagen Denmark; 2 Department of Physiotherapy and Occupational therapy University College Copenhagen Copenhagen Denmark; 3 Digital Health Department of Health Technology Technical University of Denmark Kongens Lyngby Denmark; 4 Biomedical Engineering Department of Health Technology Technical University of Denmark Kongens Lyngby Denmark; 5 Department of Geriatrics Bispebjerg and Frederiksberg Hospital Copenhagen Denmark

**Keywords:** biological age, health promotion, protocol, healthy aging, principal component analysis

## Abstract

**Background:**

Actions to improve healthy aging and delay morbidity are crucial, given the global aging population. We believe that biological age estimation can help promote the health of the general population. Biological age reflects the heterogeneity in functional status and vulnerability to disease that chronological age cannot. Thus, biological age assessment is a tool that provides an intuitively meaningful outcome for the general population, and as such, facilitates our understanding of the extent to which lifestyle can increase health span.

**Objective:**

This interdisciplinary study intends to develop a biological age model and explore its usefulness.

**Methods:**

The model development comprised three consecutive phases: (1) conducting a cross-sectional study to gather candidate biomarkers from 100 individuals representing normal healthy aging people (the derivation cohort); (2) estimating the biological age using principal component analysis; and (3) testing the clinical use of the model in a validation cohort of overweight adults attending a lifestyle intervention course.

**Results:**

We completed the data collection and analysis of the cross-sectional study, and the initial results of the principal component analysis are ready. Interpretation and refinement of the model is ongoing. Recruitment to the validation cohort is forthcoming. We expect the results to be published by December 2021.

**Conclusions:**

We expect the biological age model to be a useful indicator of disease risk and metabolic risk, and further research should focus on validating the model on a larger scale.

**Trial Registration:**

ClinicalTrials.gov NCT03680768, https://clinicaltrials.gov/ct2/show/NCT03680768 (Phase 1 study); NCT04279366 https://clinicaltrials.gov/ct2/show/NCT04279366 (Phase 3 study).

**International Registered Report Identifier (IRRID):**

DERR1-10.2196/19209

## Introduction

Healthy aging is of paramount importance when considering the trajectory of future aging populations [1,2]. Healthy aging refers to a healthy aging phenotype constituting a course of aging with high autonomy, no major chronic diseases, high quality of life, and an extended health span [3,4]. Following a healthy lifestyle earlier in life (eg, consuming alcohol moderately, not smoking, maintaining a healthy diet, and conducting regular physical activity) improves the chances of healthy aging [5,6]. Unfortunately, the steady increase in the prevalence of overweight and obesity in parallel with insufficient physical activity threatens healthy aging and emphasizes the need for effective health promotion of the general population [7-9].

Development of health literacy is a key element to promote a healthy lifestyle in the general population [10]. Applying various forms of health screenings, such as health risk assessment and health checks, is one way to track health status and thereby enable people to make qualified health decisions before diseases are manifested or progress. However, while knowledge is an important factor, it may not, by itself, motivate a change in lifestyle behavior. Health screenings often include measurements of well-established risk factors such as blood cholesterol, fasting blood glucose, and waist circumference. Although some people can understand the risk connected with these risk factors, they may be unaware of the extent to which their lifestyle affects their capability of maintaining youthful vigor and delaying morbidity to an older age. Such awareness might be pivotal and motivate changes in health behavior. Biological age plays a key role in this respect. We suggest that being “older” than stated on one’s birth certificate readily translates into disease and mortality risks, and is thus effective as health literacy to improve people’s lifestyles. In addition, we propose that biological age can be used as an outcome measure to quantify the overall placement of an individual on the healthy aging trajectory and their susceptibility to disease, which are useful in the context of primary and secondary health promotion interventions.

Unlike chronological age, biological age assesses the heterogeneity in functional metabolic status and vulnerability to disease. The increase in chronological age is uniform, whereas biological age can increase more rapidly for some and slower for others. This is due to nonmodifiable factors, such as genetics, and modifiable factors, such as lifestyle (smoking, diet, physical activity, etc) [11,12]. Biological age has been studied since the 1960s [13,14]. Much research has been directed toward finding the best biomarkers of aging [15,16] as well as the optimal method to estimate biological age [17,18]. Studies have shown that biological age can predict mortality better than chronological age and incidence of age-related diseases such as cardiovascular disease (CVD) and type 2 diabetes mellitus (T2DM) [18,19]. These results were obtained from large cross-sectional data and were derived statistically. Moreover, these studies rarely investigated the clinical use of the model in health promotion interventions.

This study aims to develop a biological age model that can distinguish between healthy and unhealthy aging among individuals with the same chronological age and sex, and investigate its clinical applicability. We apply acknowledged mathematical methodology to estimate biological age; use a combination of commonly used biological age modelling biomarkers that are minimally invasive, represent healthy aging [20], and denote the processes that change with age [15]; and explore their usefulness.

## Methods

### Overview

When developing a biological age model, it is optimal to combine knowledge of integrative physiology and health technology. Thus, our approach is interdisciplinary and involves expertise in human physiology, healthy aging, prediction modeling, and human data science.

This study protocol comprises three consecutive phases: (1) conducting a cross-sectional study to gather indicators from 100 individuals representing normal healthy aging (the derivation cohort); (2) defining a novel biological age model and estimating biological age using principal component analysis; and (3) investigating the clinical use of the model in a validation cohort of overweight adults attending a lifestyle intervention course.

### Phase 1: Derivation Cohort

#### Study Design

We recruited 100 healthy individuals equally distributed in sex and evenly spread out within the age range of 18-65 years. It is difficult to distinguish normal aging from pathological aging because physiological and functional decrements (or pathological changes) at the outset of a disease occurs as part of the normal aging process. Considering this, we excluded individuals with a history of previous or current CVD, and using medicine to reduce blood pressure, cholesterol, or glucose levels. Pregnancy is marked by physiological dynamics and is very different from the nonpregnant state (eg, the blood volume increases in the former) [21]. Thus, pregnant women or women who breastfeed were excluded from participation. In addition, people with conditions that would prevent them from enduring the cycle exercise and strength tests (eg, knee osteoarthritis) were also excluded. The study was approved by the Regional Ethics Committee, Copenhagen, Denmark (H-18031350) and was performed in accordance with the Helsinki Declaration. The study was recorded as a clinical trial (NCT03680768).

#### Candidate Biological Age Model Biomarkers

Eligible women and men arrived at the laboratory for a 2-hour examination after fasting overnight and abstaining from vigorous exercise in the prior 24 hours. The examination involved measuring 50 parameters to assess the health of the participants and collecting candidate biomarkers for the biological age model. Thus, the examination included measures of anthropometrics, physiological and metabolic health, and physical capacity as well as answering quality of life and daily physical activity questionnaires.

When selecting candidate biomarkers for the biological age model, we focused on variables that (1) characterize features of the healthy aging phenotype [20], (2) are associated with aging and age-related diseases, (3) are affected by lifestyle, and (4) are possible to obtain in a variety of settings (ie, that are not limited to use in a research setting).

Due to the mathematical approach used to estimate biological age, binary/discrete variables (eg, quality of life and education level) were not considered for the biological age model although we recognize that some of these variables are important for assessing social and mental wellbeing in the healthy aging phenotype [3].

**Figure 1:**
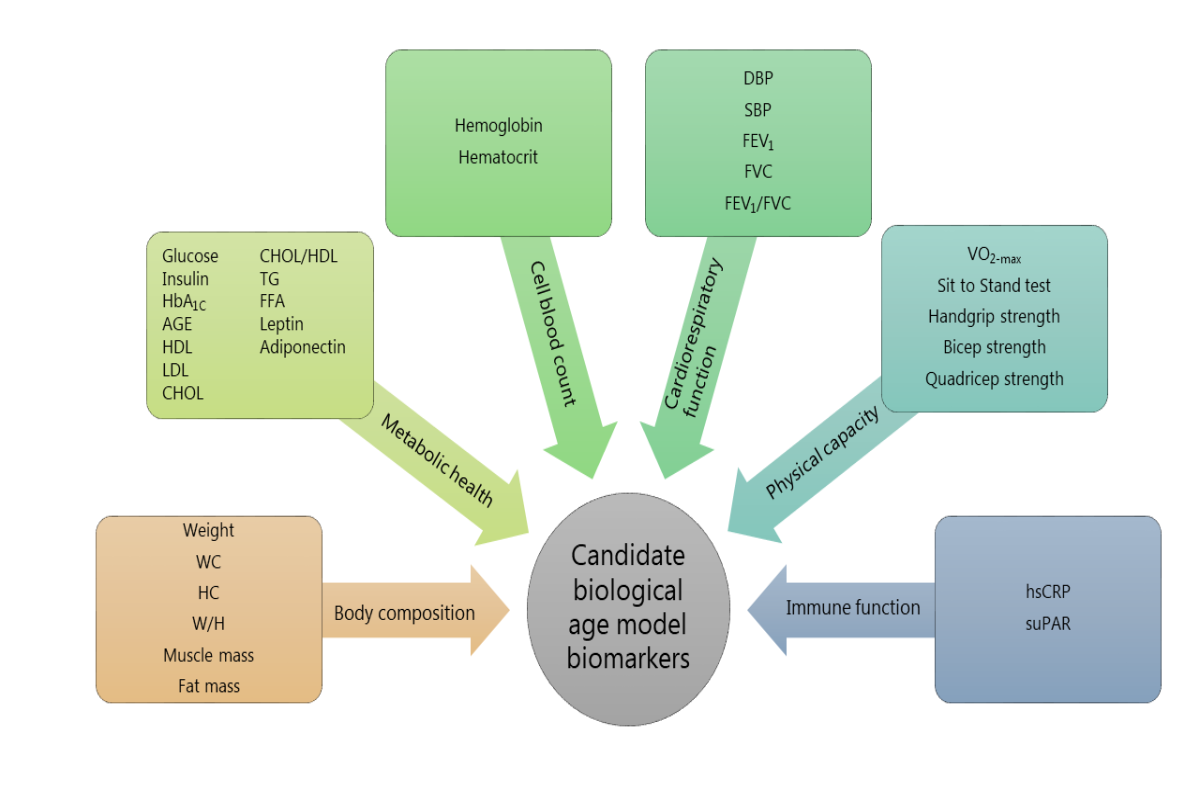
Candidate biomarkers proposed for the BA model. Each square represents 1 of the 6 following domains: (1) Body composition, (2) metabolic health, (3) cell blood count, (4) cardiorespiratory function, (5) physical capacity, and (6) immune function. The candidate biomarkers for the BA model are listed within each domain. AGE: advanced glycation end products; CHOL: Total cholesterol; CHOL/HDL: HDL to CHOL ratio; DBP: Diastolic blood pressure; FEV_1_: Forced expiratory volume within 1 second; FEV_1_/FVC: FEV_1_-FVC ratio; FFA: Free fatty acids; FVC: Forced vital capacity; Hb_A1c_: Glycated hemoglobin; HC: Hip circumference; HDL: High-density lipoprotein; hsCRP: High-sensitive C-reactive protein; LDL: Low-density lipoprotein; SBP: Systolic blood pressure; TG: Triglycerides; suPAR: soluble urokinase Plasminogen Activator Receptor; VO_2max_: Maximal oxygen uptake; WC: Waist circumference; W/H: waist to hip ratio.

In total, 32 variables were selected as candidate biomarkers and categorized in the following 6 domains: (1) body composition, (2) metabolic health, (3) cell blood count, (4) cardiorespiratory function, (5) physical capacity, and (6) immune function (Figure 1).

#### Relevance of Domains

In this section, we outline the variables included as possible biomarkers for the biological age model. We describe the variables and explain their relevance in a model that assesses healthy aging.

#### Body Composition

Aging is associated with loss of muscle mass and strength (sarcopenia) and an increase in fat mass and central adiposity. Muscle mass has been reported to begin showing a negative association with age as early as 27 years [22], with the decline in strength exceeding that in muscle mass [23]. This characteristic is related to loss in muscle quality, gradual muscle denervation, loss of type 2 muscle fibers, reduced muscle capillary density, reduced oxidative capacity, and fat infiltration [24,25].

Excess fat mass, and especially fat distribution, are important risk factors for the development of CVD and T2DM. Waist circumference and hip to waist ratio are used as surrogate measures for central adiposity and visceral adipose tissue [26,27].

#### Metabolic Health

Aging and unhealthy lifestyle are associated with reduced glucose homeostasis [28]. Fasting blood glucose concentration, HbA_1c_, and insulin sensitivity are markers of glucose homeostasis and are associated with incidence of CVD, T2DM, and mortality [20]. The prevalence of metabolic syndrome (a cluster of risk factors for T2DM and CVD) increases with age [29,30]. According to the International Diabetes Federation, the risk factors of metabolic syndrome are central obesity and any two of the following: raised triglyceride concentrations, reduced high-density lipoprotein concentrations, raised blood pressure, and raised fasting plasma glucose concentration [31]. The increase in metabolic syndrome prevalence observed with aging is associated with the age-related redistribution of fat, particularly increased central adiposity. Low levels of adiponectin are induced by visceral fat accumulation, recognized as a risk factor for CVD and T2DM, and associated in an inverse correlation with insulin resistance [32,33]. Leptin regulates the appetite, and high levels of leptin induced by subcutaneous fat accumulation may indicate decreased leptin sensitivity in obese individuals [34]. Finally, high levels of free fatty acids associated with obesity contribute to the development of peripheral insulin resistance [35].

Advanced glycation end products (AGEs) are a result of the nonenzymatic reactions between sugars and amino groups such as proteins and lipids [36]. As some AGEs have typical fluorescence bands [37], skin autofluorescence can be used as a robust noninvasive biomarker of AGE accumulation in tissues [38]. AGEs accumulate with age in healthy individuals and have been observed to accumulate faster in people with diabetes and inflammatory diseases [39]. AGEs can predict the severity of complications in diabetes [40]. The inclusion of skin autofluorescence in the Finnish Diabetes Risk Score improved the ability to detect undiagnosed diabetes and reclassify people in the intermediate risk category [41].

#### Cell Blood Count

A decrease in blood hemoglobin with age and anemia in older people is associated with functional and cognitive impairment as well as mortality [42,43]. In addition, studies on biological age modelling often include hemoglobin and hematocrit as biomarkers of aging due to their correlation with age [18,44]. Therefore, we included hemoglobin and hematocrit as candidate variables for the biological age model despite the notion that anemia is not a physiological finding related to aging per se but is associated with nutrient-related iron deficiency or unexplained anemia [45].

#### Cardiorespiratory Function

Blood pressure is a biomarker of cardiovascular (CV) function and is one of the most important modifiable risk factors that strongly predicts CV morbidity and mortality [46]. High blood pressure is a common medical condition, and its prevalence increases with age [47]. As excess fat mass represents the major factor predisposing high blood pressure, lifestyle interventions targeting obesity (and smoking) are highly relevant [48]. Forced expiratory volume in 1 second (FEV_1_), forced vital capacity (FVC), and the FEV_1_-FVC ratio are biomarkers of dynamic lung function [49]. FEV_1_ declines in a nonlinear manner with age, with the estimated decline of 25-30 mL/year starting at the age of 35-40 years and increasing up to 60 mL/year after the age of 70 years; however, the interindividual variability can be considerable [49].

#### Physical Capacity

A main indicator of physical activity and cardiorespiratory fitness is maximal oxygen uptake (VO_2max_). Functional independence is dependent on VO_2max_ [50], and its association with mortality and morbidity of noncommunicable diseases is well established [5]. Aging is associated with a decline in VO_2max_ (about 6.2%/decade) [51], primarily due to a reduction in maximal cardiac output and, secondly, due to a reduced oxygen extraction capacity at the muscle level (maximal arteriovenous oxygen difference) [52,53]. Importantly, the decline in VO_2max_ is decelerated in trained compared to sedentary subjects [54]. Finally, physical inactivity accelerates secondary aging by reduction in VO_2max_, skeletal muscle strength, and bone mineral density [55]. A Norwegian study found that sedentary time increased by 4.4 and 3.2 min/day for women and men, respectively from the age of 65 years, concomitant with a decrease in both low and moderate to vigorous physical activity [56]. Handgrip strength is a robust measure of overall strength, which correlates with mortality and declines in a linear manner with age (0.34 and 0.65 kg/year for women and men, respectively) [57,58]. Knee extension and elbow flexion are associated with functional independence and health. They are important for daily activities and have been used in previous epidemiological health investigations [59,60]. The sit to stand test is part of the “Short Physical Performance Battery” [61], which assesses lower extremity function and predicts disability in older age [59,62].

#### Immune Function

Adipose tissue is an endocrine organ and a major regulator of inflammation [63]. Excess adipose tissue is an important contributor to the elevated C-reactive protein (CRP) concentrations observed in obese people [64] and is related to the production of interleukin 6 (IL-6) and its stimulation of hepatic CRP production [65]. Chronically elevated levels of pro-inflammatory markers such as IL-6 and tumor necrosis factor-α are also key features of the aging phenotype defined as “inflammaging” [66]. Chronic low-grade inflammation (LGI) is thought to be part of the T2DM [67], CVD [68], cancer [69], and Alzheimer disease [70] pathophysiologies. CRP is considered a gold standard biomarker of low-grade inflammation and chronic inflammation. Recently, soluble urokinase plasminogen activator receptor (suPAR) was proposed as a biomarker of inflammation and was shown to predict T2DM, CVD, and cancer independently of CRP [71]. Plasma suPAR concentration increases with aging and unhealthy lifestyles (eg, unhealthy dieting and smoking) [71,72].

#### Measurements and Procedure

The examination was conducted in the order described below. Arterial blood pressure was measured in triplicate in the supine position using an automatic monitor (BoSo Medicus Control, BOSCH + SOHN GmbH). Venous blood samples were obtained for measuring concentrations of total cholesterol, high- and low-density lipoproteins, triglycerides, glucose, insulin, adiponectin, glycated hemoglobin (HbA_1c_), hematocrit, hemoglobin, CRP, and suPAR. Body composition was assessed by dual-energy X-ray absorptiometry scanning and visceral fat measurements using the CoreScan software (Lunar Prodigy Advanced, Lunar). Body composition was also assessed by bio-impedance (MC-780MA, Tanita Corporation of America Inc), which is commonly used in clinical settings. Measures of waist and hip circumference were collected. A high-quality portrait picture was taken for a subanalysis on perceived age. Skin autofluorescence was measured by an AGE Reader (DiagnOptics BV). Lung function was assessed in terms of FEV_1_ and FVC (Vyntus SPIRO spirometer, Vyaire Medical). We tested three isometric strength measures. The first test involved measuring knee extension strength. The participant was made to sit on a table. The test was performed with one leg, with the knee in 90° flexion serving as the starting position while the thigh was stabilized against the table with a standard gait belt so that it could not be lifted during the test. A standardized belt stabilization configuration was used to position the dynamometer (microFET2, Hoggan Health Industries) against the back of the table leg using a flat attachment. This method has been validated against the “gold standard” isokinetic dynamometer [73]. The second test involved measuring handgrip strength. Keeping the arm by the side, the participant was asked to squeeze a handgrip dynamometer (Takei Digital Hand Grip Dynamometer, Takei Scientific Instruments Co, Ltd). The third test measured bicep strength. The participants were asked to keep both arms by the side and flex both elbows by 90° using a Takei TKK 5402 Digital Back Strength Dynamometer (Takei Scientific Instruments Co, Ltd, Tokyo). Participants performed a minimum of 3 test trials and continued until no increase in strength occurred. A graded exercise test (Quark PFT Ergo, Cosmed) was conducted to determine VO_2max_ with an electromagnetically braked cycle ergometer (Lode Excalibur, Groeningen). The exercise protocol consisted of 5 minutes of warm-up time at 50 and 100 W for females and males, respectively, followed by a 25 W increase in load every minute until voluntary exhaustion. Finally, the participants filled out the quality of life (SF-12v2 Health Survey) and Physical Activity Score (PAS 2.1) questionnaires [74], and their education level and smoking habits were recorded.

### Phase 2: BA Estimation

#### Mathematical Approach

The three most common approaches to estimate biological age are (1) multiple linear regression (MLR) [14,75-78]; (2) principal component analysis (PCA) [19,44,79-82]; and (3) Klemera and Doubals’ method (KDM) [83,84]. Each method has its own benefits and limitations and has been compared substantially in the literature [17,18,85]. The MLR method is considered the basic approach to estimate biological age but is criticized for over- and underestimating biological age at each end of the age spectrum and the risk of biomarker multicollinearity. The PCA method derives from MLR but uses the first principal component from the PCA to form the biological age equation. This reduces the over- and underestimation observed in the MLR method and resolves the risk of multicollinearity [79]. In comparison with the MLR and PCA approaches, the KDM is a comprehensive mathematical approach. The biological age estimation is based on minimizing the distance between *m* regression lines and *m* biomarker points within an *m*-dimensional space of all included biomarkers [83]. Although the biological age estimated by the KDM has been shown to predict mortality better than that estimated by MLR and PCA [18], the majority of the studies on biological age models using minimally invasive biomarkers (essential for the use of a biological age model in health promotion) have been conducted using PCA [86]. Therefore, we will use PCA in our model development. Doing so will also allow a wider comparison of our results against more data and the findings of prior studies that had applied this approach to their models, thus facilitating an evaluation of the external validity of our model.

PCA was originally proposed by Nakamura et al [79] to select the fewest possible physiological variables to estimate biological age. Biological age construction when applying PCA includes (1) selection of the variables using correlation analysis, redundancy assessment, and loss of informative value caused by internal consistency among the variables; (2) use of PCA to obtain the principal components; (3) application of the first principal component to develop the normalized biological age score; and (4) transformation of the normalized biological age score into biological age expressed in years so that it is comparable with the chronological age [79,86]. The mathematical and statistical analysis will be completed using SAS Enterprise Guide 7.1 and MATLAB R2018b.

### Phase 3: Validation Cohort

#### Study Design

We intend to recruit overweight and obese subjects as obesity increases the risk of age-related diseases early in life. Thus, individuals with obesity are expected to deviate from the pathway of a healthy aging phenotype, resulting in a higher biological age compared to chronological age. Recruitment for the study will commence at a Danish folk high school conducting lifestyle interventions. We seek to recruit 80 overweight or obese adults (≥18 years) attending a 15-week lifestyle intervention course. Pregnancy, history of CVD, and using β-blockers are the exclusion criteria for participation in the study. The aim of the lifestyle intervention is an 8%-10% weight loss. Initial moderate weight loss induces improvements in most CV risk factors [87,88]. Therefore, this setting will allow us to explore the clinical relevance of the biological age model in assessing healthy aging. The intensive lifestyle intervention includes key features to achieve healthy aging and compress morbidity. Daily activities from 7 AM to 4 PM include supervised training (1-3 hour/day), class-based theoretical teaching focusing on changes to healthy behavior, and individual cognitive therapy. Participants are served healthy hypocaloric diets, individually prepared in accordance to an energy balance required for a normal BMI of 25 kg/m^2^. For more information on the intensive lifestyle intervention, refer to the work of Dandanell et al [89].

#### Measurements and Procedure

The results from the PCA will determine the measures to be included in the protocol. The procedure will be similar to the one described in the Phase 1 study, with the exception that we will use the short version (4 generic items) of the International Physical Activity Questionnaire and a modified exercise protocol to assess VO_2max_. To ensure that the exercise protocol elicits a valid VO_2max_, warm up will be performed at 30 and 50 W for women and men, respectively, and thereafter increased by 20 and 25 W every minute until exhaustion for women and men, respectively. Biological age will be estimated at the beginning and end of the course based on the results of the PCA. In addition, we will estimate the metabolic syndrome and Framingham risk score in the validation cohort [90,91]. Doing so will allow us to evaluate the response variation in biological age after an expected moderate weight loss and improved aerobic capacity, and we will compare these results with the changes observed in the existing validated health metrics used in health promotion and disease prevention (Framingham risk score and metabolic syndrome) [92,93]. Furthermore, we will (1) compare the biological age results in the healthy study population (the derivation cohort) with the overweight study population (the validation cohort) and (2) evaluate our biological age model against existing models to assess the feasibility of the former for health promotion.

## Results

### Phase 1

The derivation cohort consists of 51 women and 49 men. The distributions of their demographic and clinical characteristics are presented in Tables 1 and 2.

**Table 1:** Characteristics of study participants in the derivation cohort.

Variables		Women (n=51)	Men (n=49)
**Age groups (years), n (%)**			
	18-23	7 (13.7)	6 (12.2)
	24-29	7 (13.7)	6 (12.2)
	30-35	6 (11.7)	7 (14.3)
	36-41	6 (11.8)	6 (12.2)
	42-47	6 (11.8)	6 (12.2)
	48-53	6 (11.8)	6 (12.2)
	54-59	7 (13.7)	6 (12.2)
	60-65	6 (11.8)	6 (12.2)
**BMI (kg/m^2^), n (%)**			
	<25	33 (64.7)	27 (55.1)
	≥25	13 (25.5)	21 (42.9)
	≥30	5 (9.8)	1 (2.0)
HbA_1c_^a^ (mmol/mol), mean (SD)		32.3 (3.2)	33.4 (3.1)
Lung function - FEV_1_/FVC (%), mean (SD)		79.4 (6.0)	79.1 (5.3)
**Physical activity^b^ (min/week), n (%)**			
	≥150	41 (80.4)	46 (93.9)
	<150	10 (19.6)	3 (6.1)
**Education^c^ (years), n (%)**			
	<10^d^	0 (0.0)	3 (7.5)
	10-12^e^	32 (69.6)	29 (72.5)
	≥13^f^	14 (30.4)	8 (20)
**Smoking status,** **n** **(%)**			
	Yes	3 (5.9)	3 (6.1)
	No	48 (94.1)	46 (93.9)

^a^HbA_1c_: Hemoglobin A_1c_.

^b^Leisure-time spent on moderate (5 metabolic equivalents) and vigorous (6 metabolic equivalents) physical activity.

^c^Level of education was reported by 86.0% (86/100) of the total study population (46/100, 46.0% women; 40/100, 40.0% men).

^d^Lower secondary education.

^e^Upper secondary education.

^f^First- and second-stage tertiary education.

**Table 2:** Maximal oxygen consumption of study participants in the derivation cohort.

Age group (years)	VO_2max_^a^ (mL/min/kg), mean (SD)	
	Women (n=51)	Men (n=49)
18-23	36.9 (7.3)	45.2 (4.0)
24-29	37.4 (2.5)	44.8 (7.8)
30-35	37.7 (5.6)	47.9 (7.8)
36-41	36.0 (7.8)	43.2 (7.7)
42-47	39.7 (7.4)	47.1 (6.5)
48-53	29.8 (4.7)	42.7 (6.0)
54-59	30.1 (3.9)	39.7 (9.8)
60-65	31.8 (4.6)	40.1 (4.1)

^a^VO_2max_: Maximal oxygen consumption.

The majority of the participants reported having an upper secondary education (eg, high school diploma; women: 32/46, 69.6%; men: 29/40, 72.5%). Very few within the cohort (6/100, 6% in total) smoked. Cardiorespiratory fitness (VO_2max_) was moderate to high in women and men throughout the age range, the majority (women, 41/51, 80.4%; men, 46/49, 93.9%) adhering to the national recommendations of a minimum of 150 min/week of moderate to vigorous physical activity [94]. No indications of decreased lung function or T2DM were found.

**Figure 2:**
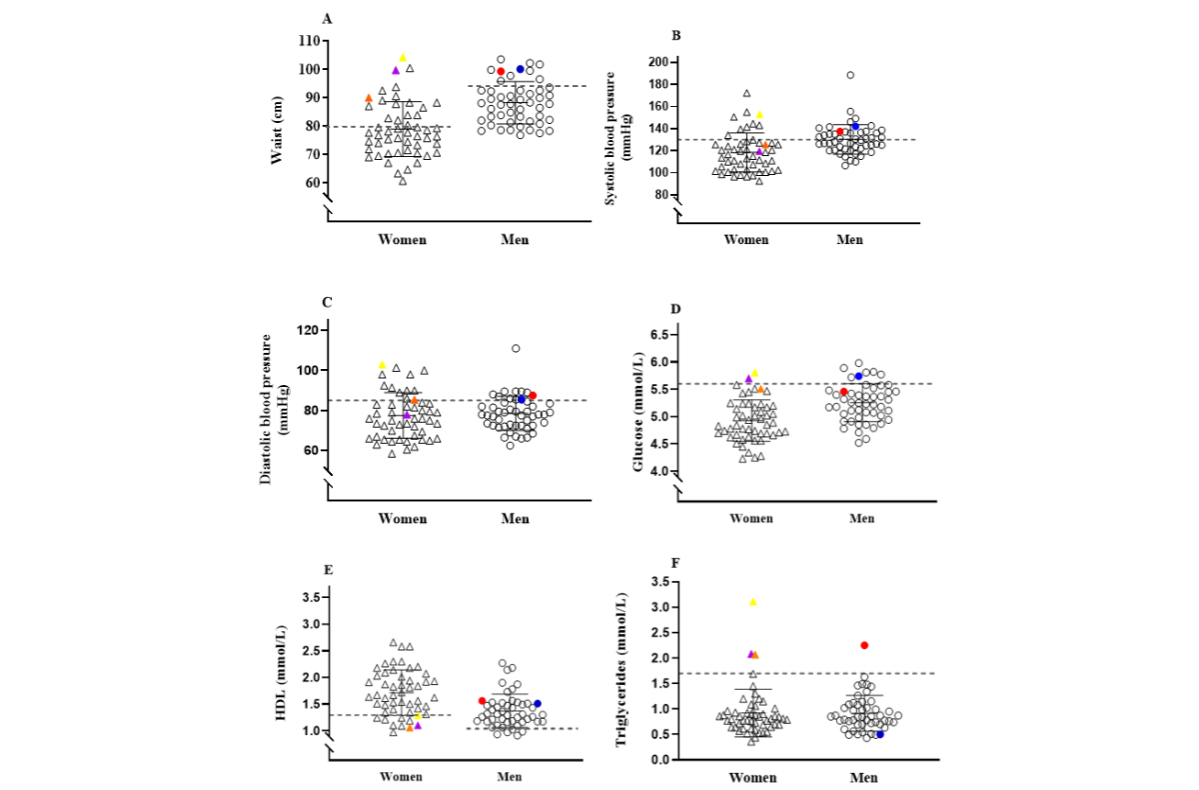
Health profile in relation to metabolic syndrome variables. The triangles represent women, and the circles represent men. Three women (yellow, orange, and purple triangles) and two men (red and blue circles) fulfilled the criteria for metabolic syndrome. The solid lines represent the mean (SD) for each group. The dashed lines represent the cut-off criteria (values mentioned in the brackets that follow) for each variable in accordance with the definition provided by the International Diabetes Federation. A: Waist circumference in females (≥80 cm) and males (≥94 cm); B: Systolic blood pressure (≥130 mm Hg); C: Diastolic blood pressure (≥85 mm Hg); D: Fasting plasma glucose (≥5.6 mmol/L); E: High-density lipoprotein (HDL) for females (˂1.29 mmol/L) and males (˂1.03 mmol/L); F: Triglycerides (≥1.7 mmol/L).

Although free from diseases, we found variations in metabolic health when assessing the cohort in terms of metabolic syndrome. Metabolic syndrome was present in 3 women and 2 men. The distribution of risk factors related to metabolic syndrome are visualized in Figure 2. We used the definition provided by the International Diabetes Federation to assess metabolic syndrome [31].

### Phase 2

Correlation analysis and principal component analysis have been performed. Interpretation of the model, including sensitivity analysis, internal consistency reliability, and model refinement, will follow.

### Phase 3

This study has been approved by the Local Research Ethics Committee, Copenhagen, Denmark (H-19073643; Clinical Trial Number NCT04279366). We have established collaboration with the staff at the folk high school and recruitment for participation is forthcoming.

## Discussion

### Findings

The primary objective of this pilot study was to develop a biological age model that could be applied for health promotion of the general adult population, given its ability to distinguish healthy and unhealthy aging trajectories among individuals with the same chronological age and sex. Within this objective lies the practical limitation of including as few and minimally invasive biomarkers in the model as possible despite the complexity of aging. Therefore, to develop a reliable biological age model, it is essential to select biomarkers that accurately show significant change with age, reflect the aging status independent of disease, have high reproducibility, cover essential areas of human function, and are appropriate for in vivo studies of humans [15,79,95]. A limitation of this study is that this biological age model is designed to assess a healthy aging trajectory only on a physical level; the assessment of the cognitive aspects of maintaining functional independence for a socially active life, an important part of the healthy aging phenotype, are not included herein [4]. Another limitation is that while the biomarkers included in the proposed biological age model align with the phenotypic biomarkers of aging (eg, clinical measures such as grip strength and glucose concentration), the model overlooks the molecular-based biomarkers of aging (ie, DNA-related markers). Short telomere length is associated with risk of CVD, age-related decline in physical function, and mortality [96]. Furthermore, DNA methylation, a biomarker for biological age (DNAm age, also referred to as the “epigenetic clock”), predicts all-cause mortality independent of the classic risk factors (age, body mass index, smoking, etc) as well as frailty, self-related health, and chronological age [96]. While such models seem promising, the lack of feasibility regarding use in community-based interventions is the main reason for not including these biomarkers in our biological age model. We do, however, plan to validate the biological age model against telomere length at a later time, when data from the derivation cohort become available. Our secondary objective involves investigating the usefulness of the model. Validating the model against mortality and morbidity is preferable but beyond the scope of this study. Instead, we plan to validate the clinical use of the model in Phase 3 by comparing the change in biological age against that in already validated prediction metrics commonly used in health promotion (eg, the Framingham risk score and metabolic syndrome) in relation to a lifestyle intervention. As the validation cohort is not randomly assigned from the general population, there is a risk that it might represent a selected group whose physiological state is independent of behavioral factors (eg, diet and physical activity) and biased by genetics. Regardless, the change in biological age after an intensive lifestyle intervention can provide initial evidence about the potential of the biological age model for health promoting interventions.

### Conclusions

We expect to find that the biological age model is a useful indicator of the risk of metabolic dysfunction and disease. Given future challenges, our expectation calls for further optimization of the model (eg, extending the sample size of the derivation cohort) and validation (by including hard endpoints such as mortality and morbidity).

## Abbreviations

AGEs: advanced glycation end products
CV: cardiovascular
CVD: cardiovascular disease
CRP: C-reactive protein
FEV_1_: forced expiratory volume in 1 second
FVC: forced vital capacity
Hb_A1c_: glycated hemoglobin
IL-6: interleukin 6
KDM: Klemera and Doubals’ method
MLR: multiple linear regression
PAS: physical activity score
PCA: principal component analysis
suPAR: soluble urokinase Plasminogen Activator Receptor
T2DM: type 2 diabetes mellitus
VO_2max_: maximal oxygen uptake
